# Identification of an alternative splicing signature as an independent factor in colon cancer

**DOI:** 10.1186/s12885-020-07419-7

**Published:** 2020-09-22

**Authors:** Haitao Chen, Jun Luo, Jianchun Guo

**Affiliations:** 1 Department of Orthopedic Surgery, Zhongnan Hospital of Wuhan University, Wuhan, 430071 China; 2 Department of Pathology, Zhongnan Hospital of Wuhan University, Wuhan, 430071 China; 3 Wuhan University Center for Pathology and Molecular Diagnostics, Wuhan, 430071 China

**Keywords:** Alternative splicing, Signature, Prognosis, Colon cancer

## Abstract

**Background:**

Colon cancer is a common malignant tumor with a poor prognosis. Abnormal alternative splicing (AS) events played a part in the occurrence and metastasis of the tumor. We aimed to develop a survival-associated AS signature in colon cancer.

**Methods:**

The Percent Spliced In values of AS events were available in The Cancer Genome Atlas (TCGA) SpliceSeq database. Univariate Cox analysis was carried out to detect the prognosis-related AS events. We created a predictive model on account of the survival-associated AS events, which was further validated with a training-testing group design. Kaplan-Meier analysis was applied to assess patient survival. The area under curve (AUC) of receiver operating characteristic (ROC) was performed to evaluate the predictive values of this model. Meanwhile, the clinical relevance of the signature and its regulatory relationship with splicing factors (SFs) were also evaluated.

**Results:**

In total, 2132 survival-related AS events were identified from colon cancer samples. We developed an eleven-AS signature, in which the 5-year AUC value was 0.911. Meanwhile, the AUC values at five years were 0.782 and 0.855 in the testing and entire cohort, respectively. Multivariate Cox regression displayed that the T category and the risk score of the signature were independent risk factors of colon cancer survival. Also, we constructed an SFs-AS network based on 11 SFs and 48 AS events.

**Conclusions:**

We identified an eleven-AS signature of colon cancer. This signature could be treated as an independent prognostic factor.

## Background

Colon cancer is one of the most common malignancies with a high death rate [1–3]. Despite significant development in tumor screening and treatment, the overall survival (OS) rates are still low in advanced patients [4–6]. Also, the prognosis may considerably differ in colon cancer patients with similar clinical characteristics due to the high heterogeneity [7]. Hence, unraveling the mechanism of tumor development and further uncovering novel prognostic biomarkers for prediction and therapeutic assessment is urgently required. In the past few decades, major advance has been achieved in the high-throughput technologies for colon cancer, including gene microarray, total RNA-sequence, and whole genome bisulfite sequencing [8–13]. However, these results mostly focused on the change of gene expression levels, but ignored the diversity of RNA types regulated by alternative splicing (AS).

The process of AS creates considerable biological complexity from a limited number of genes, and its disorder often leads to disease [14]. The AS changes observed in tumors may represent an independent carcinogenic process and may be related to the functional transformation of cancer [15]. Also, accumulating evidence has discovered that the aberrant AS events were highly associated with the occurrence and metastasis of some cancers [16–19]. Previous articles [20–23] had identified some AS events for the prognosis assessment of colorectal cancer. However, the contribution of AS to colon cancer is not fully understood. Also, the prognostic model in these papers lacks validation. Recently, Zhang et al. [24] also built an AS signature to predict the relapse of I-III colon cancer.

The present study aimed to identify and validate an AS signature for colon cancer based on the survival-associated AS events. The predictive values of the model were further evaluated. Additionally, the clinical relevance of this model and its regulatory relationship with splicing factors (SFs) was also assessed.

## Methods

### Data acquisition

We obtained the transcriptome data and survival data of colon cancer from The Cancer Genome Atlas (TCGA) database. SFs list and the Percent Spliced In (PSI) values for AS events were collected from the SpliceAid 2 database [25] and TCGA SpliceSeq [26], respectively. To obtain reliable information of ASs, only samples with a PSI value > 75% were included for further analysis [24]. Seven different subtypes of AS events were discovered, involving alternate acceptor site (AA), alternate donor site (AD), alternate promoter (AP), alternate terminator (AT), exon skip (ES), mutually exclusive exons (ME), and retained intron (RI).

### Prognosis-associated AS events

To exclude the influence of short-term follow-up on prognosis of colon cancer, samples without follow-up information or with follow-up less than 90 days were excluded. The function of impute.knn() with the impute package using R software (3.6.1) was used to replenish the missing data. When PSI value < 0.05 or the standard deviation of PSI value in all samples is less than 0.01, the AS data were also deleted. We carried out univariate Cox regression analysis to detect the survival-associated AS events, which were presented with the UpSet map and the volcano plot. Similarly, the first 15 AS events from the seven subtypes were displayed in the bubble chart.

### Identification of a prognostic AS signature

After data filtering, we randomly divided the remaining colon cancer samples into the training and testing cohorts. We conducted Lasso regression analysis to avoid the overfitting of the signature in the training cohort. Furthermore, multivariate Cox regression analysis was carried out to detect the ultimate prognostic AS events of the signature. The risk score was acquired according to the following formula:

$$ \mathrm{Risk}\ \mathrm{score}={\sum}_{\mathrm{i}=1}^{\mathrm{N}}\left(\mathrm{PSI}\ast \mathrm{Coei}\right) $$

In the training cohort, we randomly divided colon cancer patients into the high-risk and low-risk subgroups based on the median of the risk score. We performed Kaplan–Meier analysis to compare the OS between the high-risk and low-risk subgroups. Moreover, the time receiver operating characteristic (ROC) analysis was conducted to evaluate the prognostic signature. An area under the curve (AUC) > 0.75 was considered suitable for predictions. Also, the risk score distribution map, the survival status map, as well as the heatmap of PSI values were used to assess this AS signature.

### Validation of the signature

We applied the testing and entire cohorts to validate the reliability of the signature. Furthermore, we conducted survival analysis and ROC analysis to assess the signature. The risk score analysis of AS events was also applied to evaluate this signature. *P* < 0.05 for survival analysis and AUC > 0.6 was accepted for predictions.

### Applicability of the signature

To measure the prognostic value of the AS signature, we analyzed the clinical prognostic factors, including age, gender, the pathological stage, the T category (assessing the invasion of the tumor), the M category (assessing the distant metastasis of the tumor), the N category (assessing the lymph node metastasis of the tumor), and the risk score of the signature. In univariate and multivariate analysis, when *p* < 0.05, these factors were considered as independent prognostic variables. The relationships between the signature and the clinical features in the entire cohort were also evaluated.

### Construction of an SFs**-AS** network

We applied Spearman test to evaluate the correlation between the survival-associated AS events and SF genes. Correlation coefficient > 0.5 and *p* < 0.001 was the cutoff values. Subsequently, we developed an SFs-AS network, including the prognosis-associated AS events and related SFs. Moreover, Cytohubba plug-in was applied to identify the hub nodes based on eleven algorithms (seven global-based and four local-based methods).

## Results

### Data acquisition

**Fig. 1:**
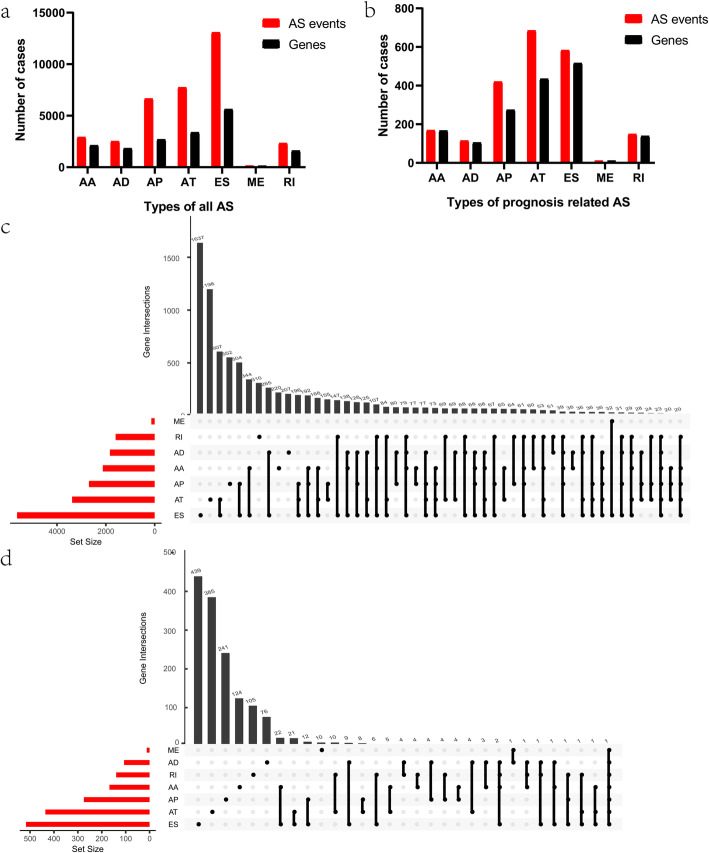
Alternative splicing (AS) events of colon cancer. **a** Numbers of all AS events and genes in 443 colon cancer patients. **b** Numbers of prognosis-related AS events and genes in 380 colon cancer patients. **c** Upset plot of all AS events. **d** Upset plot of survival-related AS events. AA, alternate acceptor site; AD, alternate donor site; AP, alternate promoter; AT, alternate terminator; ES, exon skip; ME, mutually exclusive exons; RI, retained intron

In total, 452 samples with 473 expression profiles were involved in the present study. We collected AS event profiles of 443 colon cancer samples from the TCGA SpliceSeq data portal [26]. 35,391 AS events from 17,401 genes were identified, including 7740 AT in 3381 genes, 2917 AA in 2124 genes, 2524 AD in 1833 genes, 6653 AP in 2692 genes, 13,087 ES in 5634 genes, 138 ME in 137 genes, and 2332 RI in 1600 genes (Fig. 1a). Overlaps of the seven subtypes of AS events were depicted in the UpSet plot (Fig. 1c). This indicates that one gene could own multiple kinds of mRNA splicing events. Among all these AS events of colon cancer, ME was the least common type, while ES was the most.

### Prognosis-associated AS events

**Fig. 2:**
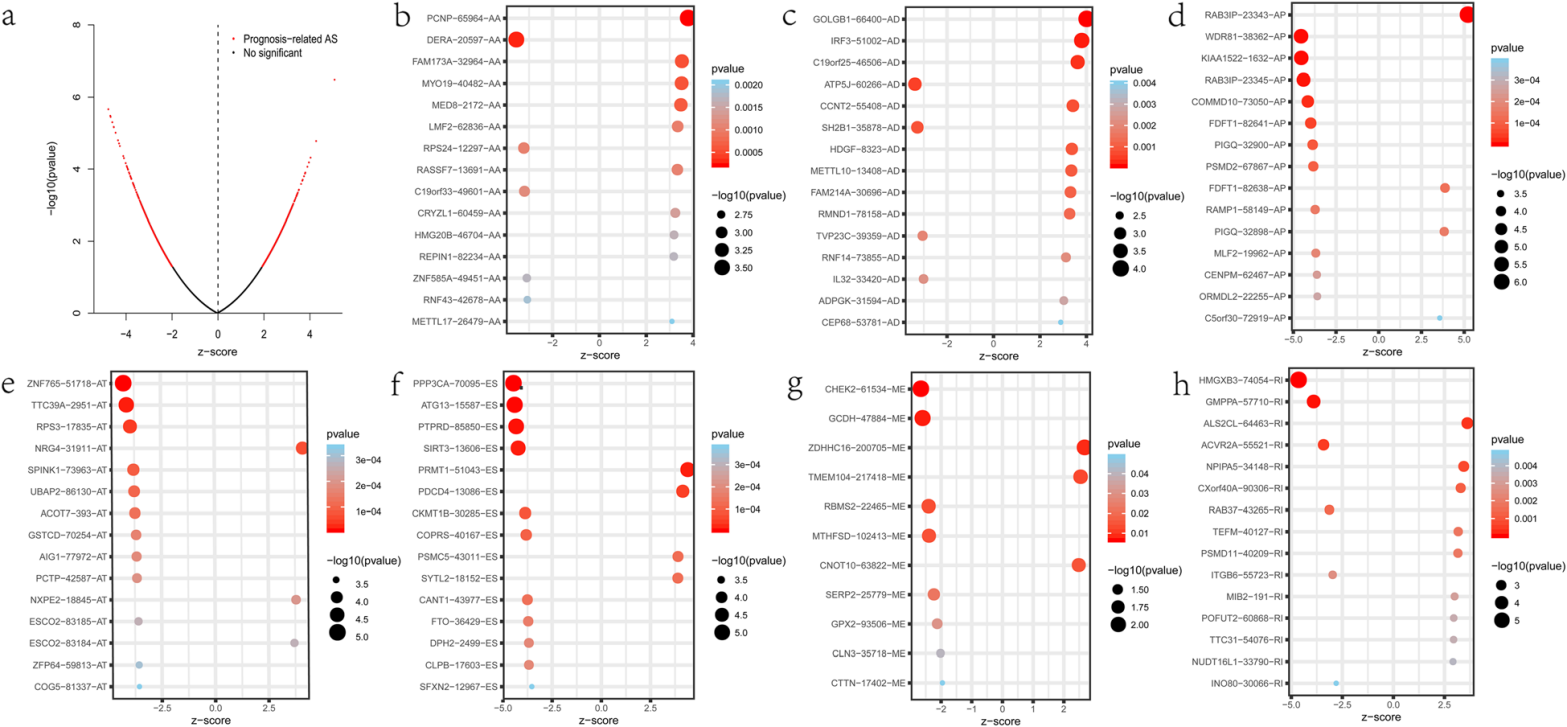
Survival-associated alternative splicing (AS) events in the colon cancer cohort. **a** The distributions of survival-related AS events in volcano plot. **b-h** Forest plots of the top 15 survival-related AS events for seven splicing subtypes

The survival-associated AS events of colon cancer were discovered by univariate Cox regression analysis. One sample lacking of follow-up data and 56 samples with follow-up less than 90 days were ruled out. Fifteen samples with a small standard deviation of the PSI values were also deleted. The prognosis-associated AS events from the remaining 380 patients were studied (Table S1). In total, 2132 AS events with 1647 genes were remarkably associated with OS (*p* < 0.05). Thus, one gene might have several AS events, among which ES was the predominant ones (Fig. 1b, d). The AS events were displayed in the volcano map (Fig. 2a). The first 15 survival-associated AS events from the seven types were exhibited in Fig. 2b-h.

### Identification of a prognostic AS signature

**Fig. 3:**
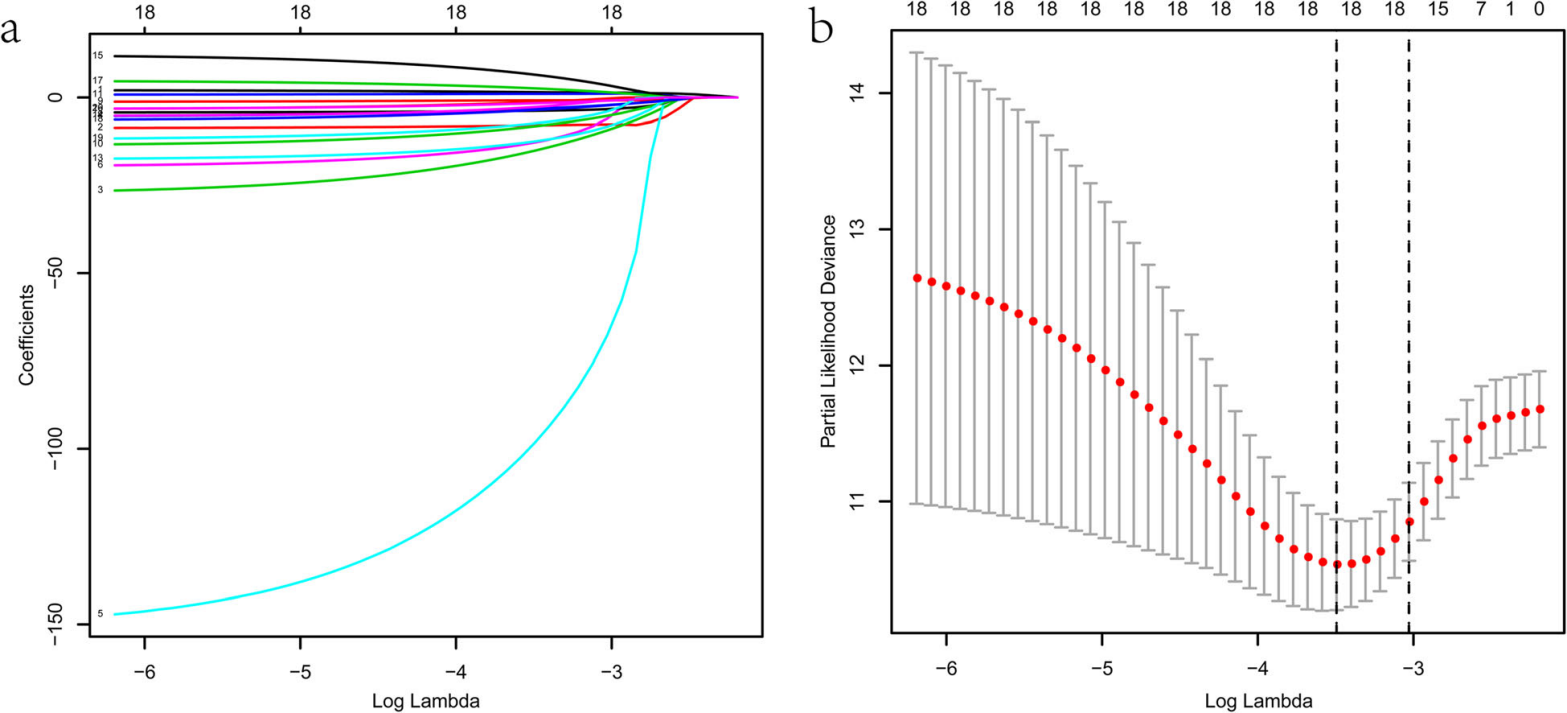
Lasso regression analysis. **a** LASSO coefficient. **b** A graph of the error rate of cross-validation

**Fig. 4:**
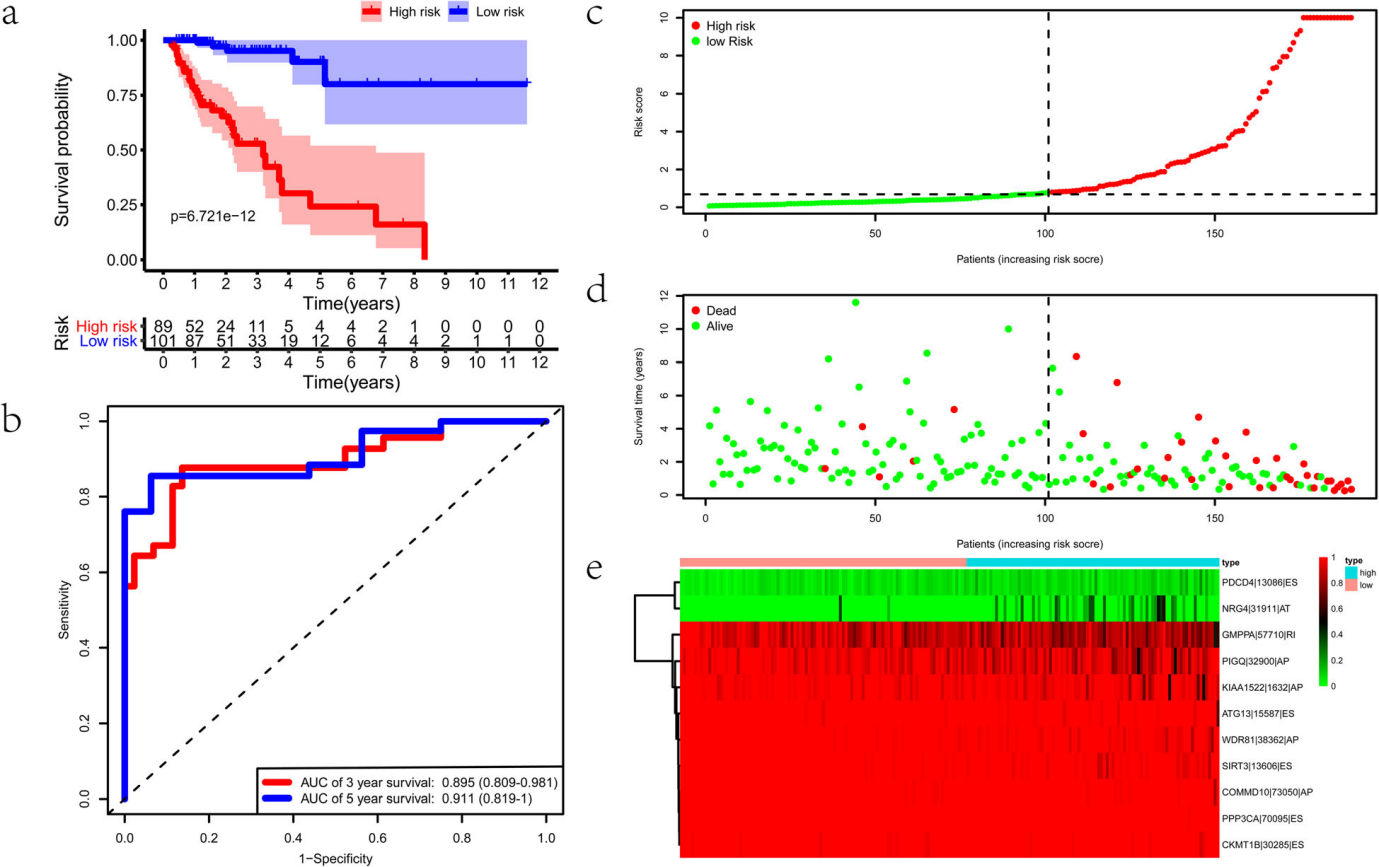
Construction of the prognostic alternative splicing (AS) signature. **a** The Kaplan–Meier plots of the prognostic signature in the training cohort. **b** The receiver operating characteristic (ROC) curves of the prognostic signature in the training cohort. **c-e** The distribution of risk score, survival status, and the PSI values of eleven AS events of each patient in the training cohort

We applied a training-testing group scheme and the 380 colon cancer samples were randomly separated into the training and testing groups (Table S2). We get eighteen-candidate prognostic AS events by conducting Lasso regression in the training group (Fig. 3a, b). Next, we carried out multivariate Cox analysis to acquire eleven optimal survival-related AS events, including WDR81–38362-AP, KIAA1522–1632-AP, PPP3CA-70,095-ES, ATG13–15587-ES, SIRT3–13606-ES, COMMD10–73050-AP, PDCD4–13086-ES, NRG4–31911-AT, GMPPA-57710-RI, CKMT1B-30,285-ES, and PIGQ-32900-AP. Among these AS events, PDCD4–13086-ES and NRG4–31911-AT are high hazard, while the remaining AS events being low hazard. The details of these prognostic AS events in the model of colon cancer were presented in Table 1. Based on the median value of the risk score, colon cancer patients were subsequently classified into the high-risk and low-risk subgroups. There was significant difference in the ROC analysis between the two groups (*p* = 6.721e-12) (Fig. 4a). The AUC values of OS for the eleven-AS events prognostic model at 3- and 5-year was 0.895 (95% confidence interval: 0.809–0.981) and 0.911 (95% confidence interval: 0.819–1), respectively (Fig. 4b). Risk score distribution map, the survival status map, and the heatmap of PSI values were shown in Fig. 4c-e.

**Table 1:** Prognostic index for colon cancer patients based on survival-related AS events

AS events	Coef	HR	HR.95 L	HR.95H	*p*value
WDR81–38362-AP	− 27.6419	9.89E-13	1.15E-16	8.48E-09	2.20E-09
KIAA1522–1632-AP	− 6.41637	0.001635	0.000139	0.019261	3.43E-07
PPP3CA-70,095-ES	− 151.93	1.04E-66	1.74E-88	6.24E-45	2.88E-09
ATG13–15587-ES	−21.3707	5.23E-10	1.28E-13	2.13E-06	4.70E-07
SIRT3–13606-ES	−19.6613	2.89E-09	1.38E-12	6.06E-06	4.68E-07
COMMD10–73050-AP	−22.1589	2.38E-10	9.79E-15	5.78E-06	1.70E-05
PDCD4–13086-ES	16.55192	15,431,416	39,384.6	6.05E+ 09	5.53E-08
NRG4–31911-AT	4.79629	121.0605	18.34399	798.9342	6.30E-07
GMPPA-57710-RI	−6.01656	0.002438	0.000161	0.03691	1.43E-05
CKMT1B-30,285-ES	−13.7515	1.07E-06	4.16E-09	0.000273	1.17E-06
PIGQ-32900-AP	−3.86593	0.020944	0.002297	0.190962	0.000608

*AS* alternative splicing

### Validation of the signature

**Fig. 5:**
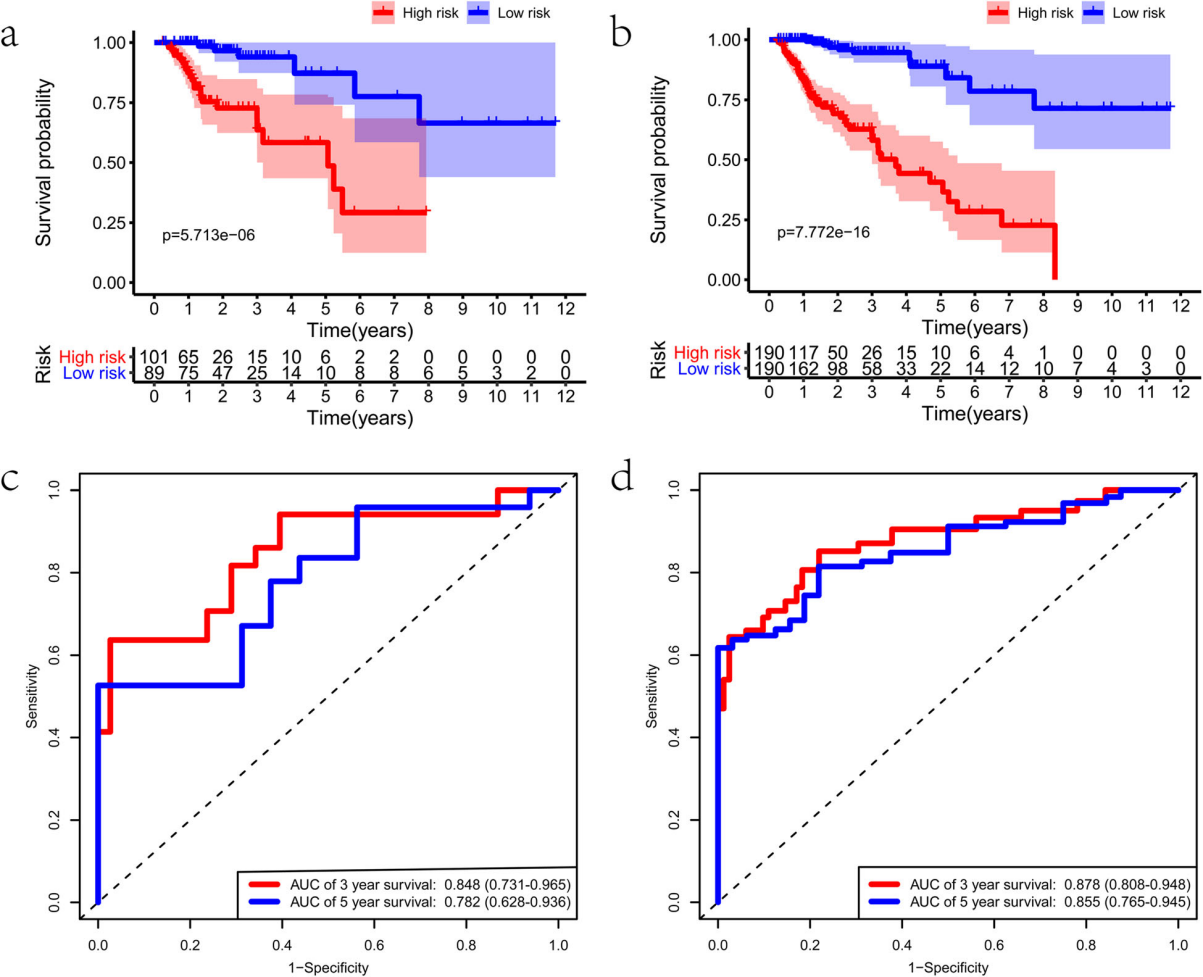
Validation of the prognostic alternative splicing (AS) signature. **a** The Kaplan–Meier plots of the AS signature in the testing cohort. **b** The Kaplan–Meier plots of the AS signature in the entire cohort. **c** The receiver operating characteristic (ROC) curves of the AS signature in the testing cohort. **d** The ROC curves of the AS signature in the entire cohort

**Fig. 6:**
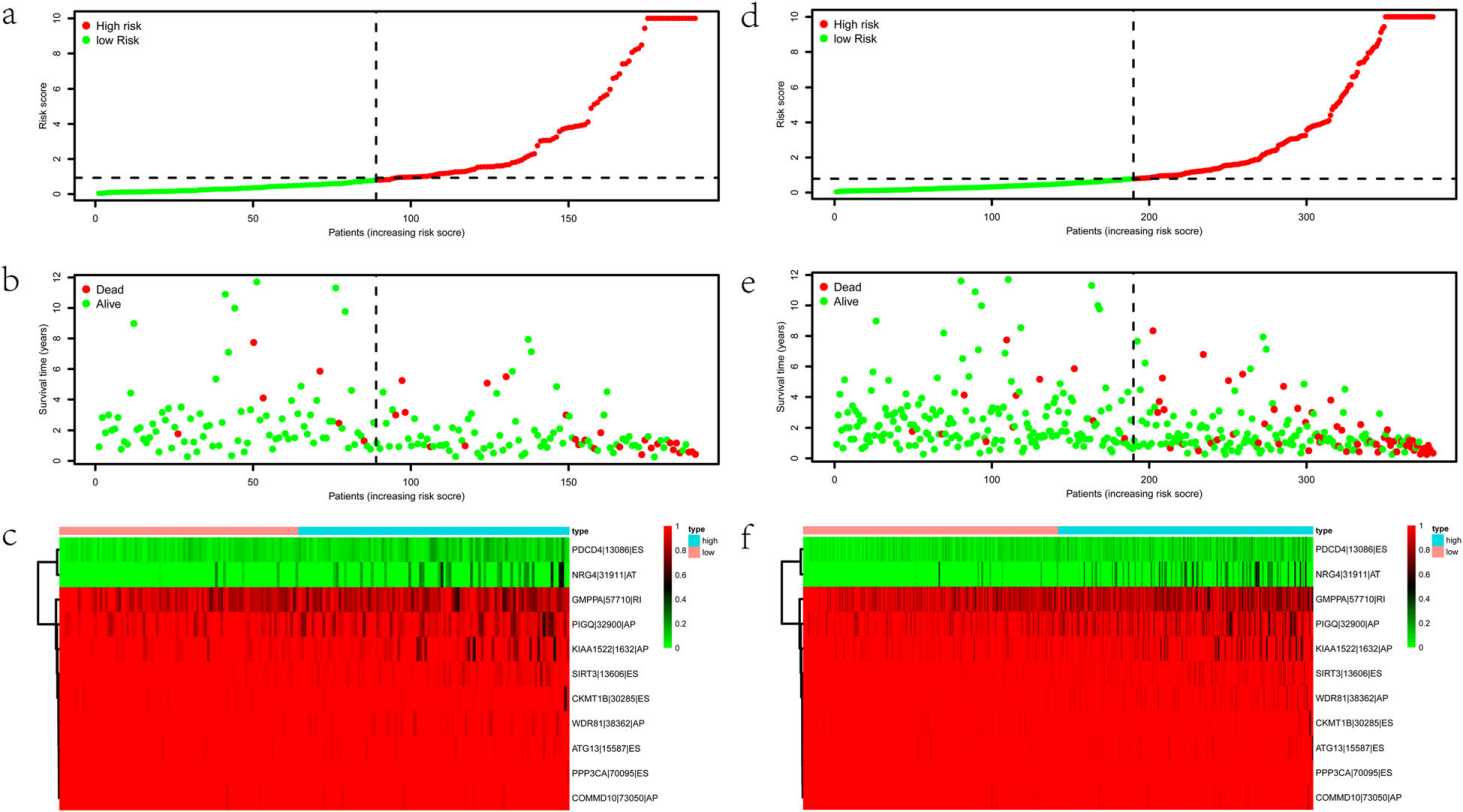
Evaluation of the prognostic alternative splicing (AS) signature. **a-c** The distribution of risk score, survival status, and the PSI values of eleven AS events of each patient in the testing cohort. **d-f** The distribution of risk score, survival status, and the PSI values of eleven AS events of each patient in the entire cohort

To confirm the usability of this signature, we validated it using the validating groups. These two validating cohorts were randomly separated into two groups on the basis of the risk score. We found significant difference in OS between the two risk groups in both cohorts (all *p* < 0.05) (Fig. 5a, b). The AUC values of both cohorts > 0.75 (Fig. 5c, d), which indicates that this signature could accurately predict the prognosis of colon cancer. Likewise, the risk curve of AS events was presented in Fig. 6a-f. All these results revealed that the AS signature was reliable in predicting the prognosis of colon cancer.

### Applicability of the signature

**Fig. 7:**
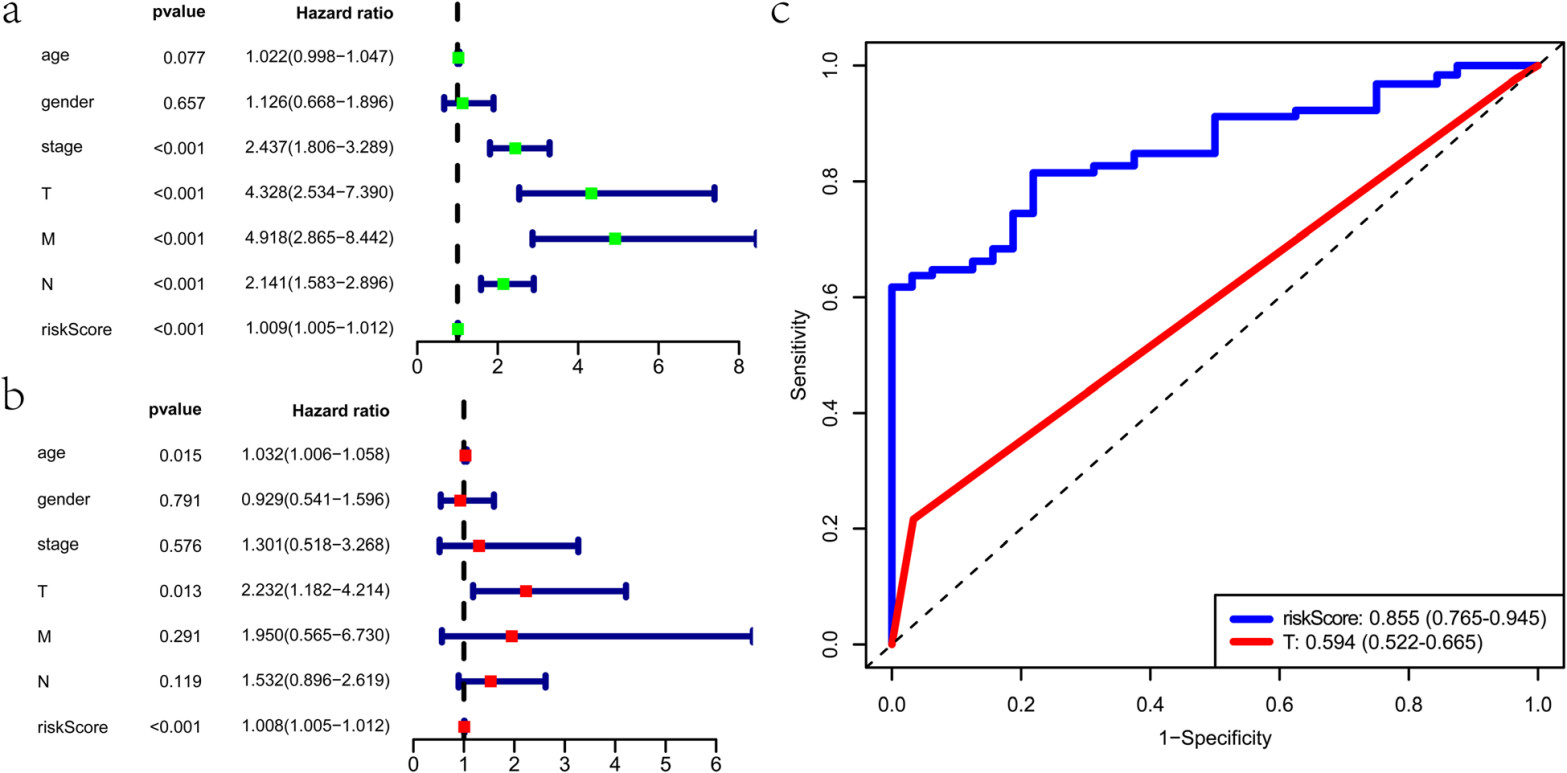
Prediction value of the prognostic signature in the entire cohort. **a** Univariate Cox analysis in the entire cohort. **b** Multivariate Cox analyses in the entire cohort. **c** The area under the curves (AUCs) at five years for the prognostic variables

Several clinical parameters, including the pathological stage, the T category, the M category, the N category, and the risk score were identified, which could predict the survival of colon cancer patients (Fig. 7a). The T category and the risk score of this signature were independent risk factors according to multivariate analysis (Fig. 7b).

**Fig. 8:**
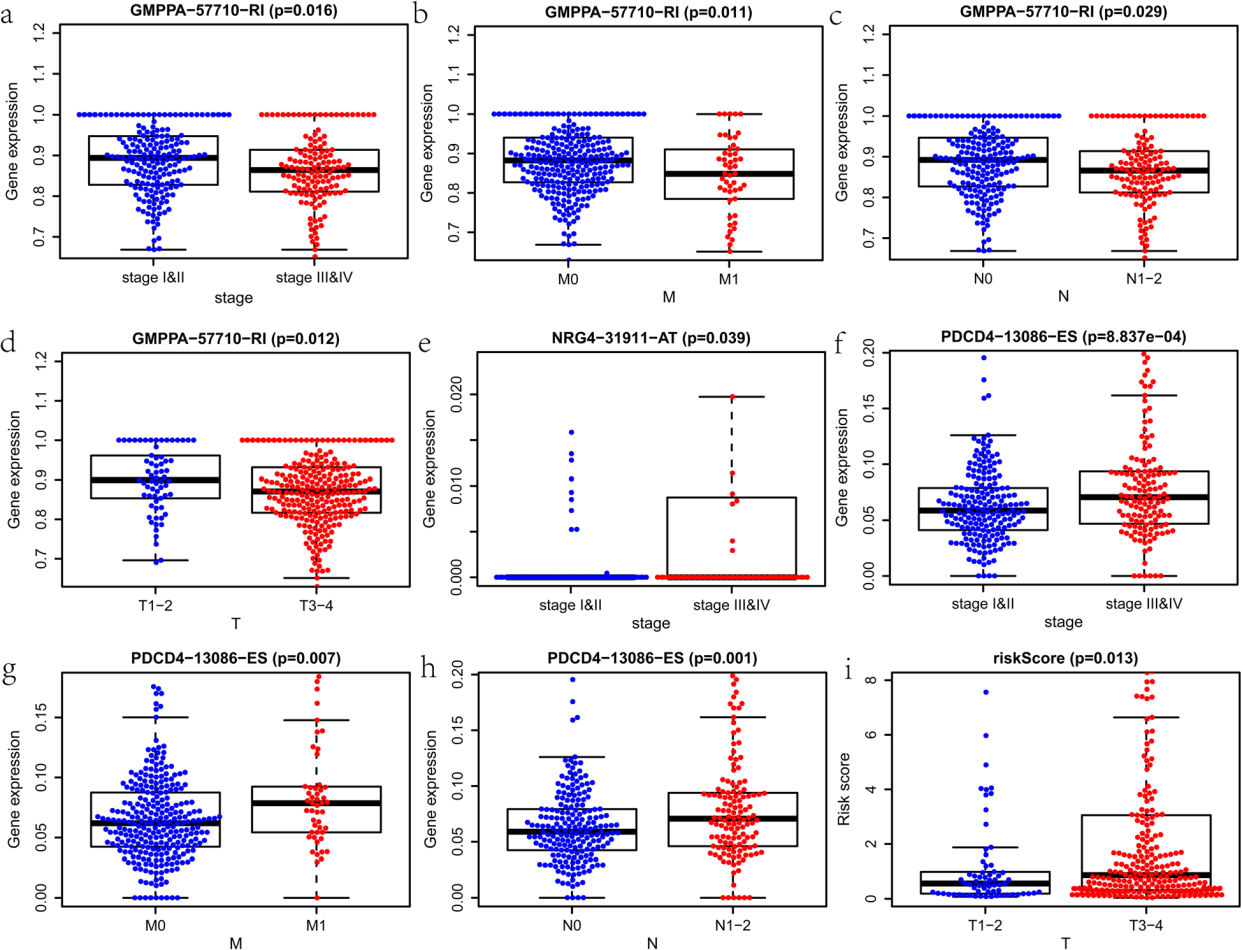
The clinical relevance of the signature in the entire cohort. **a-d** Relationship between the Percent Spliced In (PSI) value of GMPPA-57710-RI and other clinical variables, including the pathological stage, the M category, the N category, and the T category. **e** Relationship between the PSI of NRG4–31911-AT and the pathological stage. **f-h** Relationship between the PSI value of PDCD4–13086-ES and other clinical variables, including the pathological stage, the M category, and the N category. **i** Relationship between the risk score of the signature and the T category

Then, we found that the risk score of the signature was better than the T category in predicting the five-year OS (Fig. 7c). Next, we estimate the correlation of the signature with other clinical variables (Table S3). GMPPA-57710-RI was a low hazard AS event, while NRG4–31911-AT and PDCD4–13086-ES were high-hazard AS events. The PSI value of GMPPA-57710-RI was considerably lower in patients with higher pathological stage, higher M category, higher N category, and higher T category (Fig. 8a-d). On the contrary, the PSI value of NRG4–31911-AT was substantially higher in patients with a higher pathological stage. (Fig. 8e). The PSI value of PDCD4–13086-ES was markedly higher in patients with higher pathological stage, higher M category, and higher N category (Fig. 8f-h). Additionally, the risk score was higher in patients with a higher T category than those with a lower T category (Fig. 8i). These results above demonstrated that the model is a reliable and independent prognostic factor of colon cancer.

### Development of an SFs**-AS** network

**Fig. 9:**
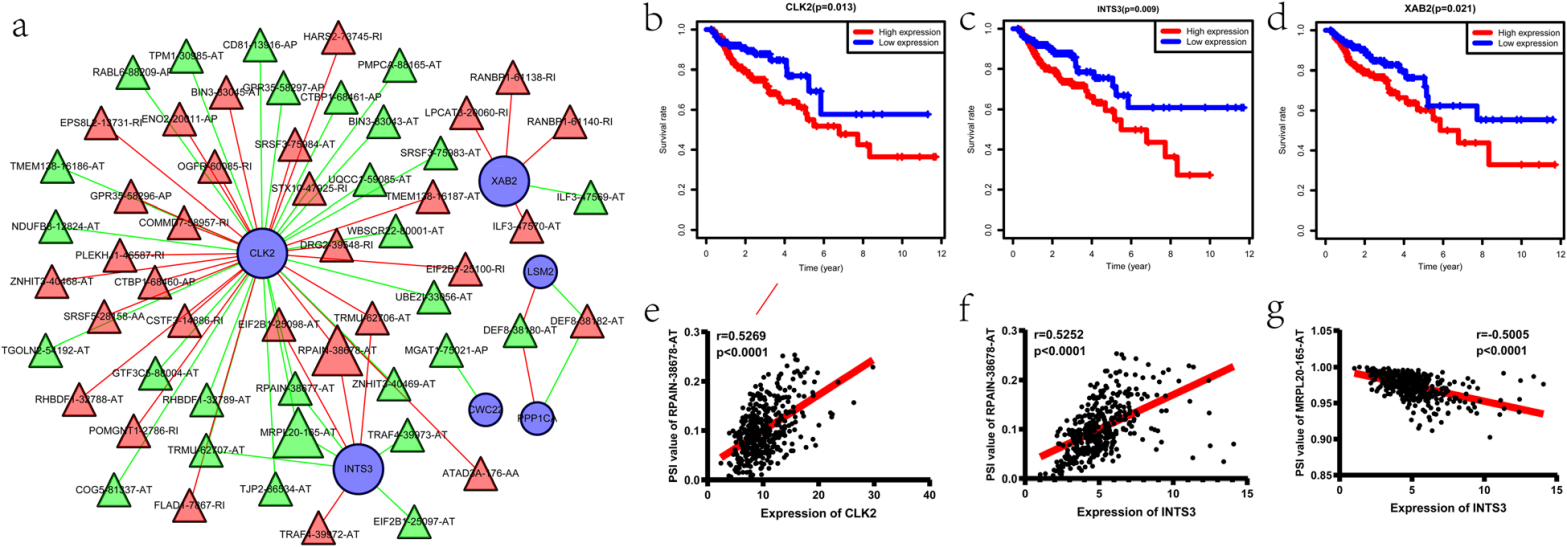
Construction of a splicing factors (SFs)-alternative splicing (AS) network. **a** The SFs-AS network. **b-d** Kaplan-Meier curves for CLK2, INTS3, and XAB2 with high (red) and low (blue) expression group in colon cancer. **e** Correlation between expression of CLK2 and PSI value of RPAIN-38678-AT. **f** Correlation between expression of INTS3 and PSI value of RPAIN-38678-AT. **g** Correlation between expression of INTS3 and PSI value of MRPL20–165-AT. For a, blue circulars represent the survival-related SFs; red triangles and green triangles represent diverse AS events and favorable AS events, respectively; red lines and green lines represent the positive and negative relationship between Percent Spliced In (PSI) values of AS events and SFs

We firstly identified 17 survival-associated SFs. The survival-associated AS events and the survival-related SFs were detected when correlation coefficient > 0.5 and *p* < 0.001, which represented a moderate correlation. Then, we rebuilt an SFs-AS network based on these survival-associated ASs and SFs, including 30 adverse AS events (red triangles), 27 favorable AS events (green triangles), and 6 SFs (blue circulars) (Fig. 9a). Several SFs, including CLK2, CWC22, INTS3, and XAB2 were linked with worse survival of patients, while LSM2 and PPP1CA were associated with favorable prognosis (Fig. 9b-d and Fig. S1). Also, we found that most favorable AS events were positively correlated with SFs of good survival, while most adverse AS events were positively associated with SFs of poor survival. Furthermore, based on the network, we detected five hub nodes, including one adverse AS event (RPAIN-38678-AT), one favorable AS event (MRPL20–165-AT), and three SFs (CLK2, INTS3, and XAB2). Correlation analysis showed that the expression of CLK2 and INTS3 were positively associated with PSI values of RPAIN-38678-AT, while the expression of INTS3 were negatively linked with PSI values of MRPL20–165-AT (Fig. 9e-g).

## Discussion

Colon cancer is a common malignant tumor with a poor prognosis. Abnormal AS events were reported to play crucial roles in the development of several cancers [27–29], which might be treated as a potential biomarker. In the present study, we detected 2132 prognosis-associated AS events from the TCGA SpliceSeq database. Then, we identified an eleven-AS signature and used the validated cohorts to evaluate the model. Next, we developed an SFs-AS network with the survival-related AS events and related SFs. Furthermore, we evaluated the prediction efficiency of the signature, which could be treated as an independent prognostic factor.

Devaud et al. [30] revealed that several AS variants of FAK might be used as potential biomarkers and treatment targets in the development and metastasis of colorectal cancer. Flodrops et al. [31] found that TIMP1 intron 3 retention could affect the progression of colon cancer. Huang et al. [32] constructed an AS signature based on differentially expressed AS events between left- and right-sided colon cancer. In the present study, we detected eleven AS events for the construction of the model. It was reported that SIRT3 silencing could be a therapeutic strategy to render colon cancer cells more sensitive to treatment [33]. In this study, SIRT3–13606-ES was a favorable AS event. This means that the ES of SIRT3 functions as a tumor suppressor in colon cancer. PDCD4 could overcome the resistance to an IGF-1R/IR Inhibitor in colon carcinoma cells, which could be used for the treatment of colon cancer [34]. In this study, PDCD4–13086-ES is a diverse AS event, which means that the ES of PDCD4 plays roles as an oncoprotein in colon cancer. The role of the remaining protein in the model of colon cancer remains not clear. However, these multiple prognosis-related AS events may partially elucidate the heterogeneity of colon cancer and contributed to the treatment of colon cancer.

Previous studies have created some molecular signatures of colon cancer. Lv et al. [35] constructed a five-lncRNA signature to predict the OS of colon cancer patients. We previously identified a five-immune gene signature for colon cancer, which contributed to its early diagnosis and prognostic prediction [36]. Similarly, Wang et al. [37] identified an epigenetic methylation-driven signature, which was associated with survival for colon cancer. In this study, we identified an eleven-AS signature of colon cancer according to the prognosis-related AS events. The AUCs of OS for this model at 5-year were larger than 0.9, which demonstrated its excellent prediction value. Additionally, the AUCs of the prognostic AS model at 5-year were larger than 0.75 in the validating cohorts, which proved the reproducibility of this model. Furthermore, the superiority of the model to other clinical parameters made this signature a better independent prognostic factor. Also, the consistence of risk score of this model with other clinical outcomes further verify the reliability of this signature.

AS changes may originate from expression changes in SFs, which affect the splicing of cancer-related genes [38, 39]. SFs could affect the specific binding of spliceosome to pre-mRNA sequences, thus generating vast and diverse mature mRNAs [14]. Furthermore, SFs function as oncoproteins or tumor suppressors and could be used as a drug target in cancer therapy [40, 41]. Chen et al. [42] demonstrated that the disordered expression levels of SFs might influence the pathogenesis of cancer. Thus, it is crucial to discover the regulatory network between the prognosis-associated AS events and the related SFs. In the present study, we constructed an SFs-AS regulatory network based on the prognosis-related AS events and SFs. We identified three hub SFs, including CLK2, INTS3, and XAB2. Until now, the role of these SFs in colon cancer is unclear. Based on our network, CLK2, INTS3, and XAB2 may function as oncoproteins because that the high expression of these SFs showed poor survival of colon cancer. In addition, these genes were positively corelated with diverse AS events. However, their potential roles in the occurrence and progress of colon cancer remains to be studied.

This study has several limitations. First, the prognostic AS signature was developed according to public databases, which needed further verification by future clinical researches. Second, the relationship between the survival-related AS events and their corresponding SFs, as well as the underlying mechanisms behind the development of colon cancer, requires further study.

## Conclusion

We identified an eleven-AS event signature. This signature could be treated as an independent prognostic factor.

## Supplementary information

**Additional file 1.**

**Additional file 2.**

**Additional file 3.**

**Additional file 4.**

## Data Availability

All analyzed data are included in this published article and its supplementary information file. The original data are available upon reasonable request to the corresponding author.

## Abbreviations

OS: Overall survival
AS: Alternative splicing
SFs: Splicing factors
TCGA: The Cancer Genome Atlas
PSI: Percent Spliced In
AA: Alternate acceptor site
AD: Alternate donor site
AP: Alternate promoter
AT: Alternate terminator
ES: Exon skip
ME: Mutually exclusive exons
RI: Retained intron
ROC: Receiver operating characteristic
AUC: Area under the curve
