# Supplementary material for: Identification of an alternative splicing signature as an independent factor in colon cancer
Source: BMC Cancer. 2020 Sep 22;20:904. doi: 10.1186/s12885-020-07419-7 (PMC7510085; doi:10.1186/s12885-020-07419-7)
Supplement: Supplementary file 1 — Additional file 1. [file 12885_2020_7419_MOESM1_ESM.docx]

**Table S1** Clinical information of the 380 colon cancer patients in the entire cohort

| Clinical Traits | Variable | N (Total=380) | Percentage (%) |
| --- | --- | --- | --- |
| Survival status | Alive | 309 | 81.3 |
|  | Dead | 71 | 18.7 |
| Age (years) | <70 | 206 | 54.2 |
|  | >=70 | 174 | 45.8 |
| Gender | Female | 172 | 45.3 |
|  | Male | 208 | 54.7 |
| Pathological stage | Stage I | 67 | 17.6 |
|  | Stage II | 145 | 38.2 |
|  | Stage III | 108 | 28.4 |
|  | Stage IV | 50 | 13.2 |
|  | Unknow | 11 | 2.9 |
| T category | T1 | 9 | 2.4 |
|  | T2 | 67 | 17.6 |
|  | T3 | 258 | 67.9 |
|  | T4 | 45 | 11.8 |
|  | TX | 1 | 0.3 |
| M category | M0 | 284 | 74.7 |
|  | M1 | 50 | 13.2 |
|  | MX | 42 | 11.1 |
|  | Unknow | 4 | 1.1 |
| N category | NO | 224 | 58.9 |
|  | N1 | 90 | 23.7 |
|  | N2 | 66 | 17.4 |
