# Supplementary material for: Identification of an alternative splicing signature as an independent factor in colon cancer
Source: BMC Cancer. 2020 Sep 22;20:904. doi: 10.1186/s12885-020-07419-7 (PMC7510085; doi:10.1186/s12885-020-07419-7)
Supplement: Supplementary file 2 — Additional file 2. [file 12885_2020_7419_MOESM2_ESM.docx]

**Table S2** Grouping of the colon cancer patients

| Clinical Traits | Variable | Training Cohort | Testing Cohort | Entire Cohort |
| --- | --- | --- | --- | --- |
| Survival status | Alive | 150 (39.5%) | 159 (41.8%) | 309 (81.3%) |
|  | Dead | 40 (10.5%) | 31 (8.2%) | 71 (18.7%) |
