# Supplementary material for: Identification of an alternative splicing signature as an independent factor in colon cancer
Source: BMC Cancer. 2020 Sep 22;20:904. doi: 10.1186/s12885-020-07419-7 (PMC7510085; doi:10.1186/s12885-020-07419-7)
Supplement: Supplementary file 3 — Additional file 3. [file 12885_2020_7419_MOESM3_ESM.docx]

**Table S3** Correlation between the model of colon cancer and the clinical features

| AS events | age | gender | stage | T | M | N |
| --- | --- | --- | --- | --- | --- | --- |
| WDR81-38362-AP | -0.293(0.770) | 0.342(0.732) | 0.879(0.380) | 0.635(0.527) | 1.419(0.162) | 0.883(0.378) |
| KIAA1522-1632-AP | -0.872(0.384) | -1.809(0.072) | 0.545(0.586) | 1.644(0.102) | 0.957(0.342) | 0.087(0.931) |
| PPP3CA-70095-ES | 1.354(0.178) | -0.518(0.605) | 0.58(0.563) | 1.352(0.177) | 0.903(0.371) | -1.353(0.178) |
| ATG13-15587-ES | 0.697(0.487) | 0.226(0.822) | -1.63(0.104) | 0.549(0.584) | -1.623(0.107) | -1.677(0.095) |
| SIRT3-13606-ES | 0.011(0.991) | 0.649(0.517) | 1.184(0.238) | 1.767(0.079) | 1.666(0.101) | 1.181(0.239) |
| COMMD10-73050-AP | 0.617(0.538) | -0.667(0.506) | 0.794(0.428) | -0.604(0.547) | 0.812(0.419) | 0.546(0.586) |
| PDCD4-13086-ES | -0.997(0.320) | -0.166(0.868) | -3.367(8.837e-04) | -1.938(0.055) | -2.779(0.007) | -3.296(0.001) |
| NRG4-31911-AT | -0.463(0.644) | -0.828(0.408) | -2.072(0.039) | -1.877(0.062) | -1.049(0.298) | -1.741(0.083) |
| GMPPA-57710-RI | -0.078(0.937) | 0.574(0.567) | 2.432(0.016) | 2.568(0.012) | 2.605(0.011) | 2.196(0.029) |
| CKMT1B-30285-ES | 1.537(0.126) | 0.292(0.771) | -0.05(0.960) | 0.703(0.483) | -0.199(0.842) | -0.333(0.739) |
| PIGQ-32900-AP | -0.748(0.455) | -1.1(0.272) | 1.711(0.088) | 1.289(0.200) | 0.398(0.692) | 1.823(0.070) |
| riskScore | -1.719(0.088) | -0.089(0.929) | -0.285(0.776) | -2.491(0.013) | -1.193(0.237) | 0.72(0.472) |

AS alternative splicing
