# Supplementary figures and images for: Identification of an alternative splicing signature as an independent factor in colon cancer
Source: BMC Cancer. 2020 Sep 22;20:904. doi: 10.1186/s12885-020-07419-7 (PMC7510085; doi:10.1186/s12885-020-07419-7)

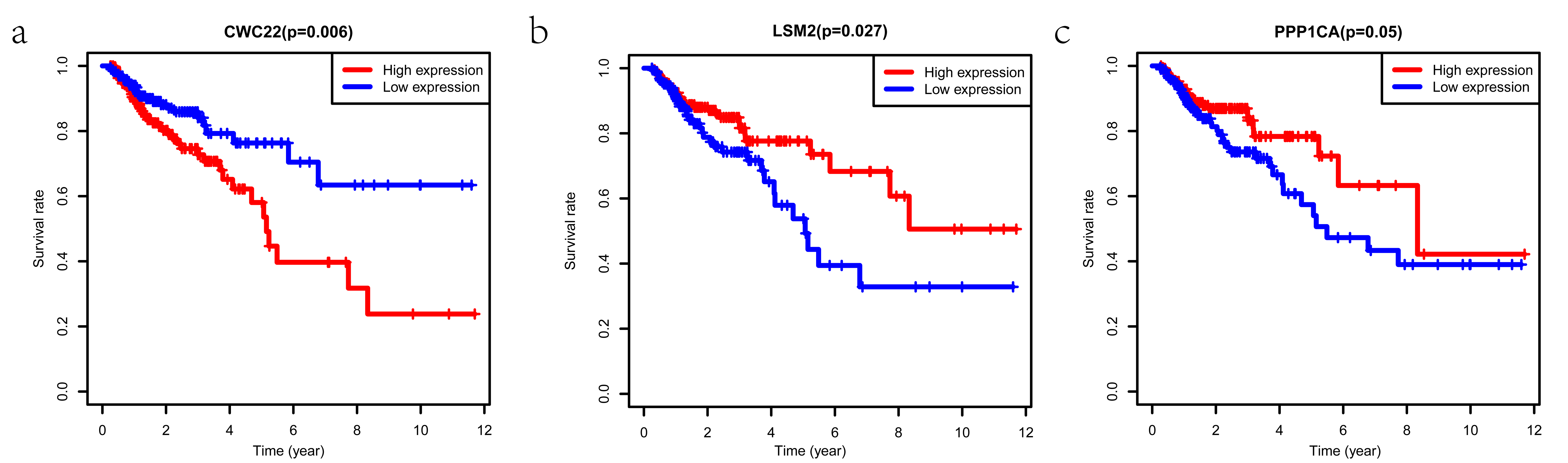

Supplement: Supplementary file 4 — Additional file 4. [file 12885_2020_7419_MOESM4_ESM.tif]
